# Supplementary material for: A self-organized synthetic morphogenic liposome responds with shape changes to local light cues
Source: Nat Commun. 2021 Mar 9;12:1548. doi: 10.1038/s41467-021-21679-2 (PMC7943604; doi:10.1038/s41467-021-21679-2)
Supplement: Supplementary file 1 — Supplementary Information [file 41467_2021_21679_MOESM1_ESM.pdf]

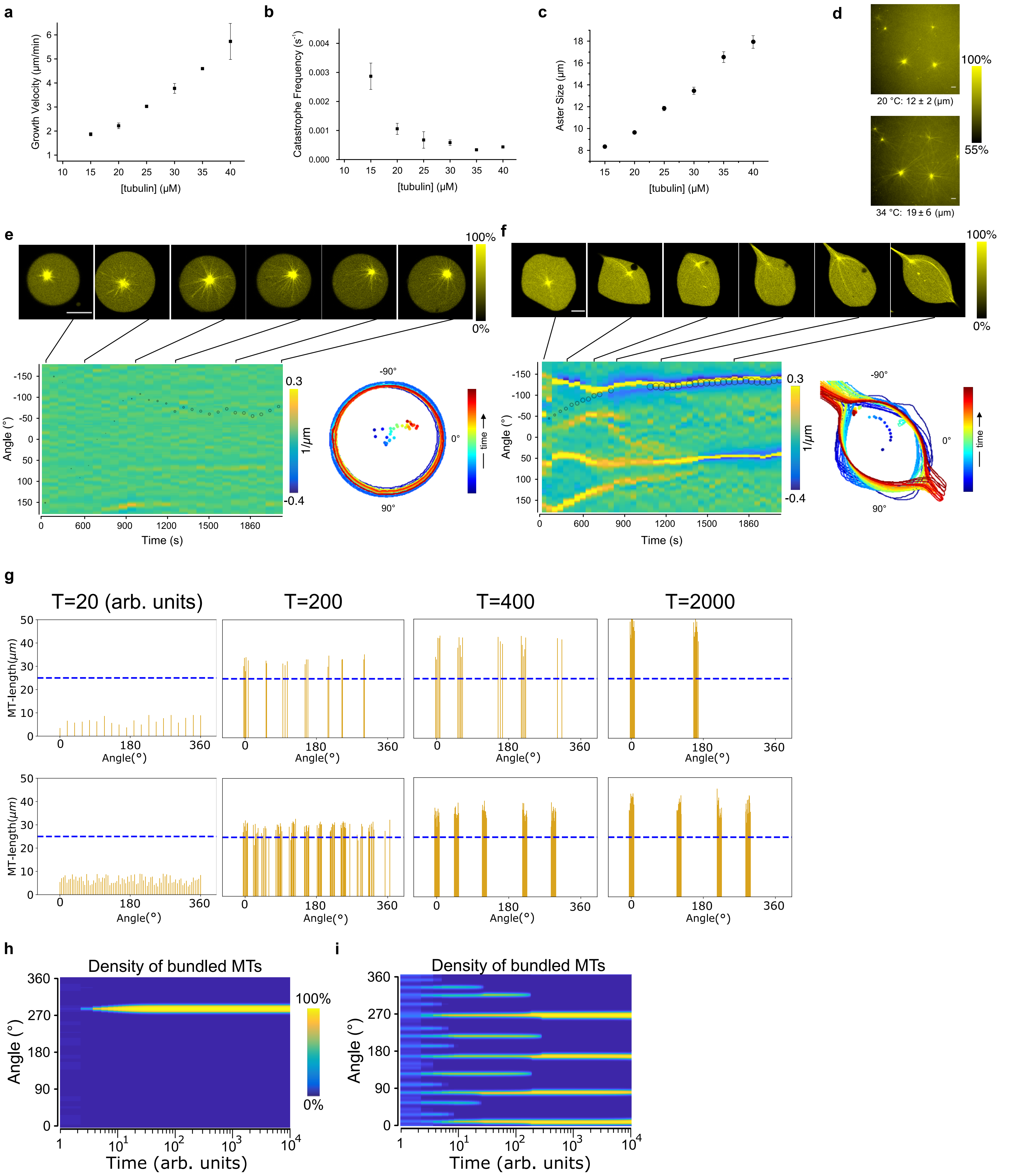

a
Growth Velocity (μm/min)
[tubulin] (μM)
b
Catastrophe Frequency (s-1)
[tubulin] (μM)
c
Aster Size (μm)
[tubulin] (μM)
d
100%
55%
20 °C: 12 ± 2 (μm)
34 °C: 19 ± 6 (μm)
e
100%
0%
Angle (°)
Time (s)
0.3
1/μm
-0.4
-90°
0°
90°
time
f
100%
0%
Angle (°)
Time (s)
0.3
1/μm
-0.4
-90°
0°
90°
time
g
T=20 (arb. units)
T=200
T=400
T=2000
MT-length(μm)
Angle(°)
h
Density of bundled MTs
Angle (°)
Time (arb. units)
100%
0%
i
Density of bundled MTs
Angle (°)
Time (arb. units)

Supplementary Fig. 1. Characterization of MT dynamics on glass surface and morpho-dynamics of encapsulated MT-asters.

Dependence of microtubule (MT)-growth velocity (a) and catastrophe frequency (b) on tubulin concentration as determined by single-filament TIRF microscopy assays (mean±S.E.M, 76 tracked filaments per condition from N=3 independent experiments). (c) Size of MT-asters on glass surface as a function of tubulin concentration as the 99% decay length from fitting the cumulative fluorescence of multiple overlaid MT-aster to an exponential function (34 per condition from N=2 independent experiments). Error bars: standard error of the regression (Methods). (d) Temperature dependence of MT-aster size. Representative CLSM images of normalized tubulin^{568} fluorescence of MT-asters (40µM tubulin, yellow color bar) before (20°C, top) and after heating to 34°C (bottom). Average radius of asters and standard error of the regression (Methods) below images. (e) Top: CLSM time-lapse of temperature change induced aster growth in a GUV with rigid membrane (yellow color bar: normalized fluorescence intensity). Bottom left: Angular membrane curvature kymograph overlaid with centrosome position (small circle: centered, large circle: membrane proximal; color bar: curvature as the inverse radius (1/µm) of an inscribed circle (Methods). Positive curvature: circle inside GUV; negative: circle outside. Lines connect to micrographs at indicated times. Bottom right: GUV contours during time-lapse, color-coded by time (colored dots: corresponding centrosome positions). (f) CLSM time-lapse of temperature change induced aster growth in a GUV with deformable membrane (yellow color bar: normalized fluorescence intensity). Bottom left: Angular membrane curvature kymograph overlaid with centrosome position as in (e). Bottom right: GUV contours during time-lapse, color-coded by time as in (e). (g) Agent-based model implemented with Monte Carlo simulations that explicitly considers the stochastic MT-dynamics (Methods, Supplementary Video 2). Snapshots of the evolution of the MT-membrane sub-system towards stable global organization of the MTs. Dashed blue line depicts the position of the membrane. Top: total MT number = 20, forming two stable protrusions at opposed poles.; bottom: total MT number = 80 forming four stable protrusions (Supplementary Video 2, right). Other parameters in Methods. (h,i) Simulation of the paradigmatic reaction-diffusion model in Fig. 1f yielding similar organization of MT bundling in dependence of total MT number as the agent-based model in (g). (h) Kymograph of bundled MT density for total amount of MTs c_1=1100 evolving toward a polar initial pattern with single MT-bundle. (i) Same as in (h) for c_1=2700 evolving towards a star-like pattern. For both simulations, the feedback strength γ_1=1 (Methods, color bar: normalized density). Scale bars: 10µm.

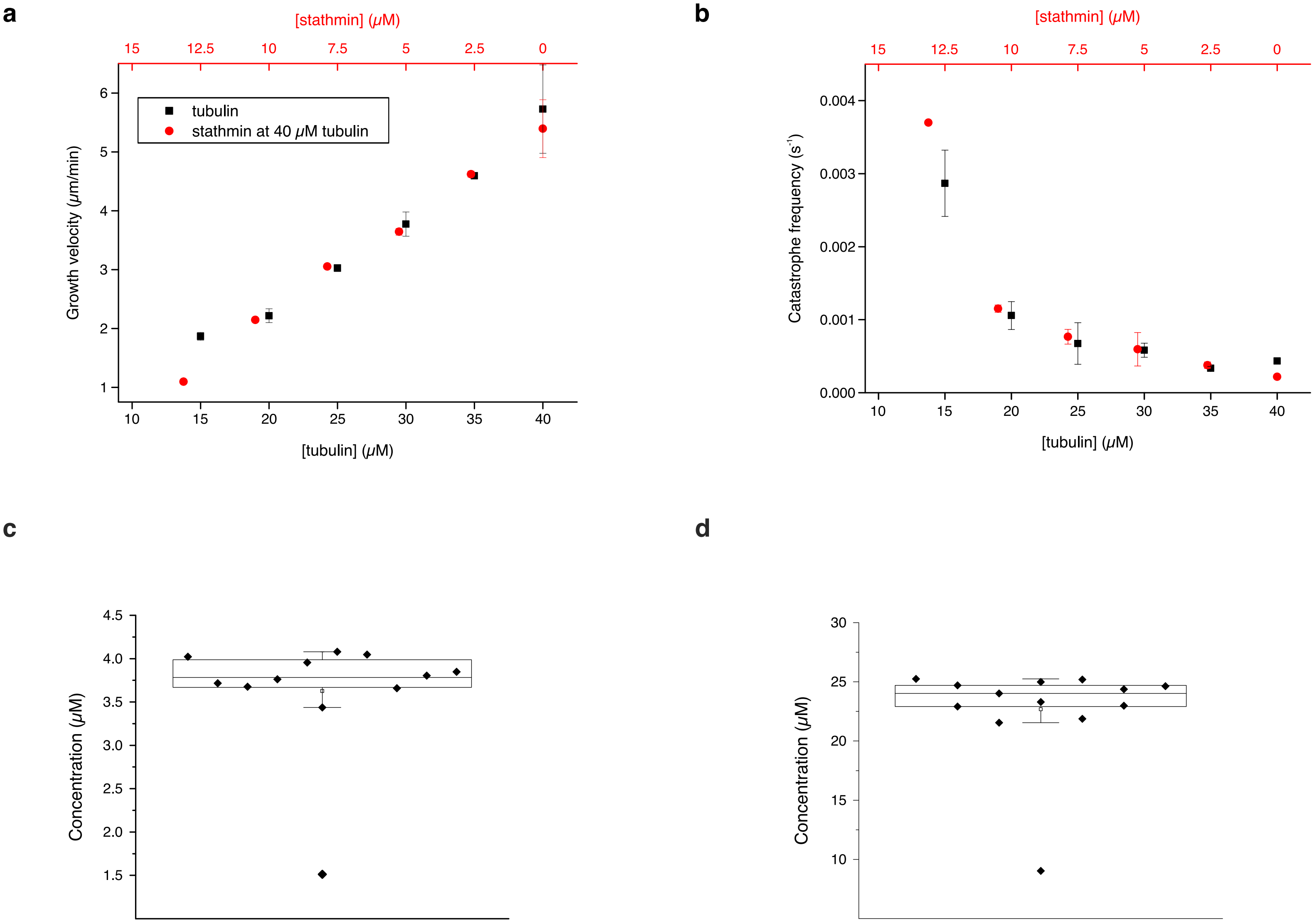
a
[stathmin] (μM)
15
12.5
10
7.5
5
2.5
0
tubulin
stathmin at 40 μM tubulin
Growth velocity (μm/min)
1
2
3
4
5
6
10
15
20
25
30
35
40
[tubulin] (μM)
b
[stathmin] (μM)
Catastrophe frequency (s-1)
0.000
0.001
0.002
0.003
0.004
[tubulin] (μM)
c
Concentration (μM)
1.5
2.0
2.5
3.0
3.5
4.0
4.5
d
Concentration (μM)
10
15
20
25
30

Supplementary Fig. 2. Regulation of MT-dynamics by stathmin and encapsulation efficiencies

(a) Dependence of MT-growth velocity, and (b) catastrophe frequency on tubulin concentration (black squares, black axis on bottom) and stathmin concentration (red dots, 40μM tubulin, red axis on top) as determined by single-filament TIRF microscopy assays (mean±S.E.M, 75 tracked filaments per condition, N=3). (c) Encapsulation efficiency of $stathmin^{647}$ in GUVs: 72±14% (encapsulation concentration 5μM, final encapsulated concentration 3.6±0.7μM, n=12). For quantification, 1μM $stathmin^{647}$ was added to the outside as reference. (d) Encapsulation efficiency of $tubulin^{568}$ in GUVs: 75±13% (encapsulation concentration 30μM, final encapsulated concentration 22.7±4μM, n=13). For quantification, 1μM $tubulin^{568}$ was added to the outside as reference. Box-plots: individual GUV (diamond), 25th and 75th percentile (box), min-max (whiskers), median (line) and mean (square).

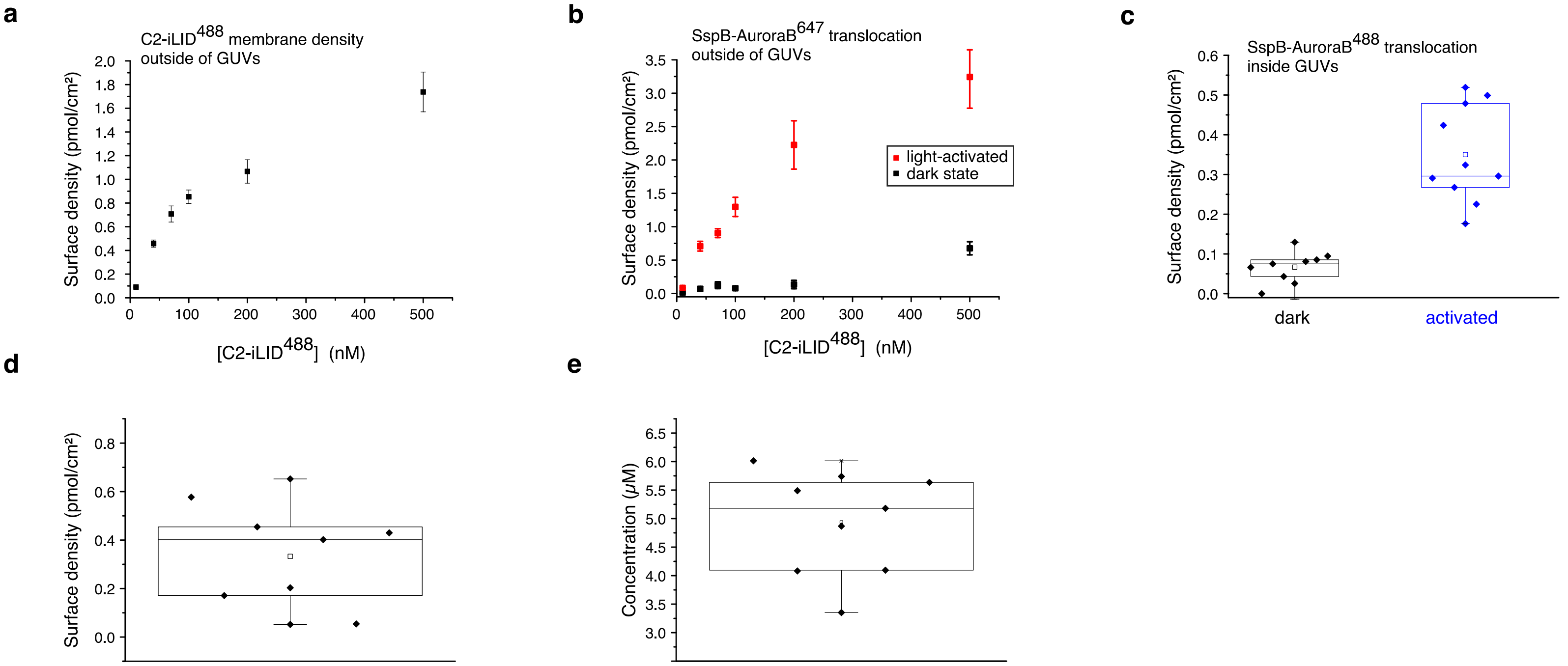

a
C2-iLID488 membrane density
outside of GUVs
Surface density (pmol/cm²)
[C2-iLID488] (nM)
b
SspB-AuroraB647 translocation
outside of GUVs
light-activated
dark state
Surface density (pmol/cm²)
[C2-iLID488] (nM)
c
SspB-AuroraB488 translocation
inside GUVs
Surface density (pmol/cm²)
dark
activated
d
Surface density (pmol/cm²)
e
Concentration (μM)

Supplementary Fig. 3. Light-induced SspB-AuroraB translocation and encapsulation efficiency of the dimerizer system

(a) Titration of C2-iLID^{488} on the outside of GUVs (mean±S.D, n=3). (b) Recruitment of SspB-AuroraB^{647} (1.5µM in bulk) to GUV membranes for different concentrations of C2-iLID^{488}, with (red) and without (black) 488nm irradiation (mean±S.D, n=3). (c) Surface density of encapsulated SspB-AuroraB^{488} on the inner leaflet of GUV membranes, before (black, n=9) and after (blue, n=10) 488nm irradiation. Encapsulation concentration of SspB-AuroraB^{488}: 3.5µM, C2-iLID: 5µM. (d) Surface density of encapsulated C2-iLID^{488} on the inner leaflet of GUV membranes from a encapsulation concentration of 3.5µM: 0.33±0.2pmol/cm^2 (n=9). 1µM soluble iLID-tRac1^{488} (Methods) was added to the outside for quantification, and the brightness ratio of the two proteins was determined separately. (e) Encapsulation efficiency of SspB-AuroraB^{488} inside GUVs: 40.8±7.5% (encapsulation concentration of 12µM gives a final encapsulated concentration of 4.9±0.9µM, n=9). For quantification, 0.3µM SspB-AuroraB^{488} was added to the outside as reference. Box-plots in c-e: individual GUV (diamond), 25th and 75th percentile (box), 1.5 interquartile range (whiskers), median (line) and mean (square).

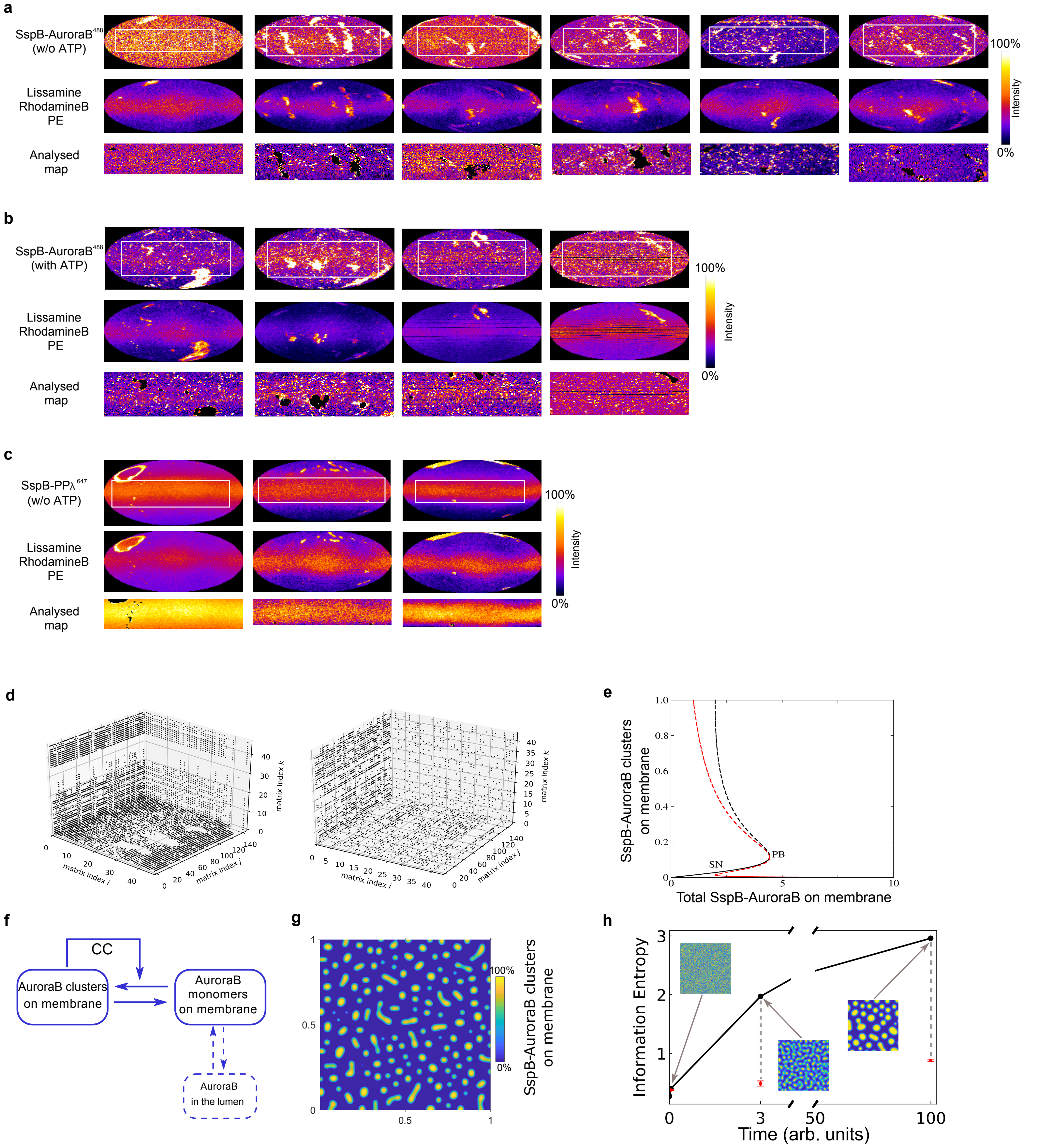

a
SspB-AuroraB488 (w/o ATP)
Lissamine RhodamineB PE
Analysed map
100%
Intensity
0%
b
SspB-AuroraB488 (with ATP)
Lissamine RhodamineB PE
Analysed map
100%
Intensity
0%
c
SspB-PPλ 647 (w/o ATP)
Lissamine RhodamineB PE
Analysed map
100%
Intensity
0%
d
matrix index i
matrix index j
matrix index k
e
SspB-AuroraB clusters on membrane
Total SspB-AuroraB on membrane
SN
PB
f
CC
AuroraB clusters on membrane
AuroraB monomers on membrane
AuroraB in the lumen
g
SspB-AuroraB clusters on membrane
100%
0%
h
Information Entropy
Time (arb. units)

Supplementary Fig. 4. Regularity of Spatial SspB-AuroraB patterns on membrane

(a) Top: fluorescence intensity maps of SspB-AuroraB^{488} encapsulated in GUVs without ATP after light-induced translocation to the membrane (warm-to-cold color bar: normalized intensity). Middle: corresponding Lissamine-RhodamineB-PE fluorescence-distribution. Bottom: maps cropped from white rectangular areas highlighted in top row, where lipid defects (thresholded from Lissamine-RhodamineB-PE fluorescence-distribution in middle row) were excluded. (b) as (a), with 2mM encapsulated ATP. (c) as (a), but for SspB-PPλ^{647} instead of SspB-AuroraB^{488}. (d) Representative spatial recurrence plots for the intensity distribution of membrane-translocated SspB-AuroraB^{488} (left) and SspB-PPλ^{647} (right), used for the quantification in Fig. 3f. Three-dimensional slices of the four-dimensional recurrence plots are represented. (e) Bifurcation diagrams of the two component SspB-AuroraB model depicted in Fig. 3h. Equations and parameters in Methods. PB – pitchfork bifurcation; SN – saddle-node bifurcation. Solid lines: black – homogenous steady state, red – symmetry broken state. Dashed lines: unstable steady states. (f) Representation of the 3-component SspB-AuroraB cluster-pattern generating mechanism. CC (cooperative clustering) causes depletion of monomers on the membrane that are translocated from the lumen. The respective pattern is shown in (g). Equations and parameters in Methods. Color bar: normalized density of clustered SspB-AuroraB. (h) Evolution of information entropy as the two-component system (Fig. 3h) progresses towards a stable pattern. Information entropy was estimated from recurrence quantification of the simulated SspB-AuroraB spatial distributions (insets). Red dots: Information entropy upon pattern randomization (3 numerical randomizations, mean±S.D. connected with dashed lines).

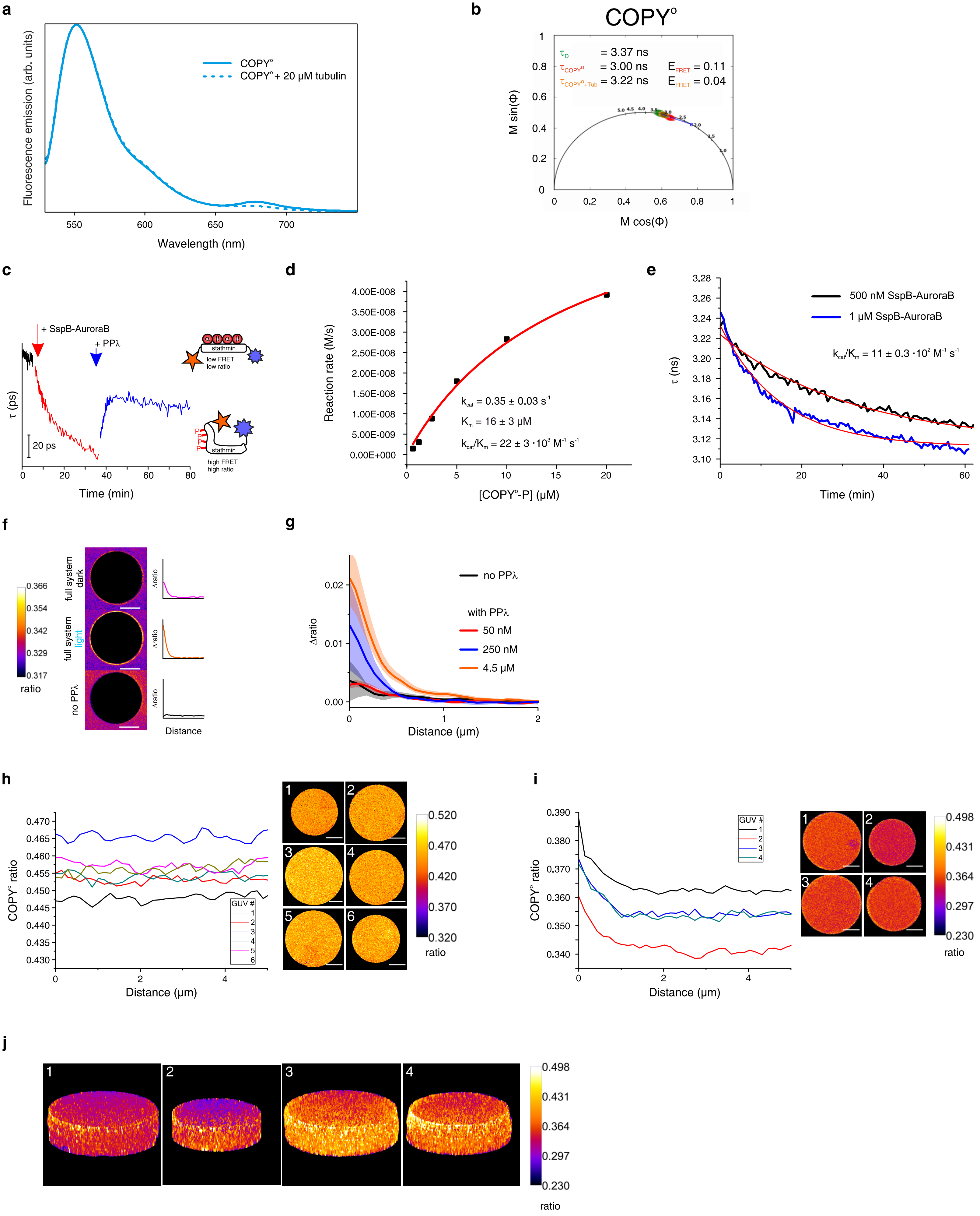
a
Fluorescence emission (arb. units)
Wavelength (nm)
COPY°
COPY° + 20 µM tubulin
b
COPY°
τD = 3.37 ns
τCOPY° = 3.00 ns EFRET = 0.11
τCOPY°+Tub = 3.22 ns EFRET = 0.04
M sin(Φ)
M cos(Φ)
c
+ SspB-AuroraB
+ PPλ
τ (ps)
20 ps
Time (min)
stathmin
low FRET
low ratio
high FRET
high ratio
d
Reaction rate (M/s)
kcat = 0.35 ± 0.03 s-1
Km = 16 ± 3 µM
kcat/Km = 22 ± 3 ·10³ M-1 s-1
[COPY°-P] (µM)
e
500 nM SspB-AuroraB
1 µM SspB-AuroraB
kcat/Km = 11 ± 0.3 ·10² M-1 s-1
τ (ns)
Time (min)
f
full system dark
full system light
no PPλ
ratio
Δratio
Distance
g
no PPλ
with PPλ
50 nM
250 nM
4.5 µM
Δratio
Distance (µm)
h
COPY° ratio
GUV #
Distance (µm)
ratio
i
COPY° ratio
GUV #
Distance (µm)
ratio
j
ratio

Supplementary Fig. 5. Spatial segregation of the stathmin (de)phosphorylation cycle generates a steady-state pStathmin gradient

(a) Fluorescence emission spectra of 1µM COPY^{o} (donor: Atto532, acceptor: Atto655) in absence (solid lines) or presence of 10µM tubulin (dashed lines) upon 520nm excitation. (b) Quantification of COPY^{o} FRET efficiency by Time-Correlated Single Photon Counting Fluorescence Lifetime Imaging Microscopy (TCSPC-FLIM). Phasor plot representation of the Fourier components of the first harmonic frequency (20MHz) of TCSPC-FLIM data is shown. Average fluorescence lifetime of the COPY^{o} construct was determined by global analysis of FLIM data. Average fluorescence lifetime of COPY^{o} without acceptor (τ_D) was 3.37±0.05ns (green). COPY^{o} exhibited a lifetime of 3.0±0.1ns with a corresponding E_{FRET} of 0.11±0.03, in the absence of tubulin (red), while tubulin binding (orange) increased the lifetime to 3.2±0.1ns with a corresponding E_{FRET} of 0.04±0.03. (c) Steady-state stathmin phosphorylation in solution by FLIM of the FRET-sensor COPY^{o} (10µM) in presence of 20µM tubulin and 2mM ATP. Enzyme addition indicated by arrows (red: 2µM SspB-AuroraB; blue: 0.5µM PPλ, final concentrations). Right: open dephosphorylated tubulin-bound conformation with high lifetime (top) and closed phosphorylated conformation with low lifetime (bottom). (d) Dephosphorylation reaction rates of COPY^{o}-P by 0.5µM PPλ with kinetic parameters from Michaelis-Menten fit (Methods, Supplementary Table 1). (e) COPY^{o2} (10µM) phosphorylation kinetics by SspB-AuroraB in the presence of 20µM tubulin. k_{cat}/K_m was estimated from corresponding monoexponential fits at two SspB-AuroraB concentrations (Supplementary Table 1). (f) Ratiometric CSLM fluorescence images of COPY^{o} (4µM) phosphorylation outside of GUVs with iLID_G (0.5µM initial concentration) on the membrane, SspB-AuroraB (12µM) and tubulin (20µM). Top: before 488nm irradiation (250nM PPλ outside of GUV). Middle: after light-induced translocation of SspB-AuroraB. Bottom: without PPλ. Right: corresponding ratiometric, baseline-subtracted profiles (Δratio). (g) Δratio profiles of COPY^{0} (4µM) with SspB-AuroraB^{488} (12µM) and C2-iLID (0.5µM) on the membrane at different PPλ concentrations outside GUVs after 488nm irradiation (mean±S.E.M., n=4 per concentration). (h) Ratiometric profiles of COPY^{o} fluorescence inside GUVs without PPλ (n=6) and corresponding CLSM fluorescence images derived from maximum intensity ratio z-stacks (8 slices at equatorial plane; final concentrations: 40±7µM tubulin, 4±1µM COPY^{o}, and 5±1µM SspB-AuroraB; 5±3 10^{-1}pmol/cm² C2-iLID. 0µm defines the membrane position. (i) As (h) with 5±1 10^{-1}µM PPλ (final concentration, n=4). (j) 3D projections of the GUVs corresponding to the ratio profiles in (i). Cold-to-warm color bar in (f), (h)-(j) denotes ratio. Scale bars: 10µm.

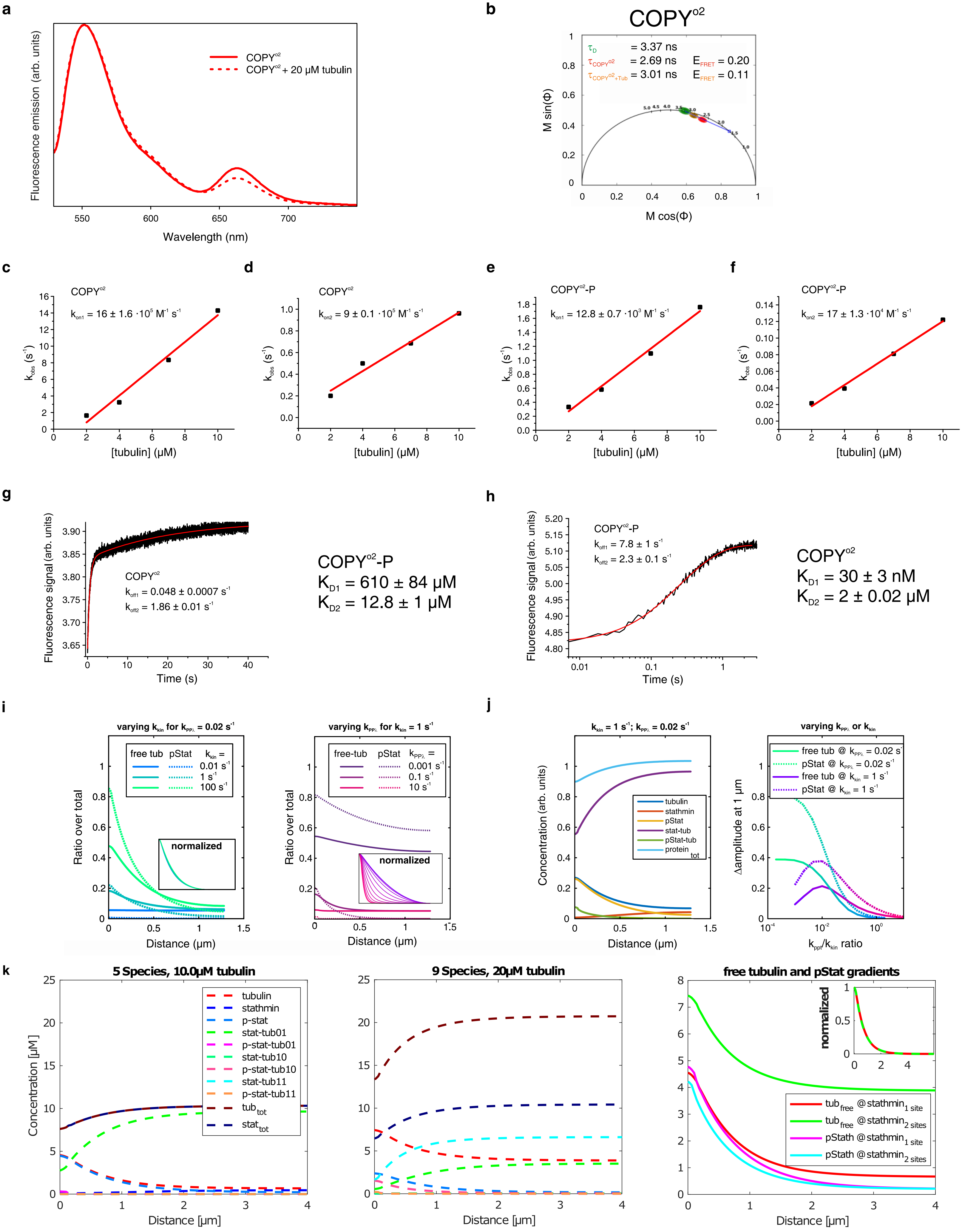

a
Fluorescence emission (arb. units)
Wavelength (nm)
COPY^o2
COPY^o2 + 20 µM tubulin
b
COPY^o2
τD = 3.37 ns
τCOPY^o2 = 2.69 ns
EFRET = 0.20
τCOPY^o2+Tub = 3.01 ns
EFRET = 0.11
M sin(Φ)
M cos(Φ)
c
COPY^o2
kon1 = 16 ± 1.6 ·10^5 M^-1 s^-1
kobs (s^-1)
[tubulin] (µM)
d
COPY^o2
kon2 = 9 ± 0.1 ·10^5 M^-1 s^-1
kobs (s^-1)
[tubulin] (µM)
e
COPY^o2-P
kon1 = 12.8 ± 0.7 ·10^3 M^-1 s^-1
kobs (s^-1)
[tubulin] (µM)
f
COPY^o2-P
kon2 = 17 ± 1.3 ·10^4 M^-1 s^-1
kobs (s^-1)
[tubulin] (µM)
g
Fluorescence signal (arb. units)
COPY^o2
koff1 = 0.048 ± 0.0007 s^-1
koff2 = 1.86 ± 0.01 s^-1
Time (s)
COPY^o2-P
KD1 = 610 ± 84 µM
KD2 = 12.8 ± 1 µM
h
Fluorescence signal (arb. units)
COPY^o2-P
koff1 = 7.8 ± 1 s^-1
koff2 = 2.3 ± 0.1 s^-1
Time (s)
COPY^o2
KD1 = 30 ± 3 nM
KD2 = 2 ± 0.02 µM
i
varying kkin for kPPλ = 0.02 s^-1
free tub pStat kkin =
0.01 s^-1
1 s^-1
100 s^-1
normalized
Ratio over total
Distance (µm)
varying kPPλ for kkin = 1 s^-1
free-tub pStat kPPλ =
0.001 s^-1
0.1 s^-1
10 s^-1
normalized
Ratio over total
Distance (µm)
j
kkin = 1 s^-1; kPPλ = 0.02 s^-1
tubulin
stathmin
pStat
stat-tub
pStat-tub
protein tot
Concentration (arb. units)
Distance (µm)
varying kPPλ or kkin
free tub @ kPPλ = 0.02 s^-1
pStat @ kPPλ = 0.02 s^-1
free tub @ kkin = 1 s^-1
pStat @ kkin = 1 s^-1
Δamplitude at 1 µm
kppt/kkin ratio
k
5 Species, 10.0µM tubulin
tubulin
stathmin
p-stat
stat-tub01
p-stat-tub01
stat-tub10
p-stat-tub10
stat-tub11
p-stat-tub11
tub tot
stat tot
Concentration [µM]
Distance [µm]
9 Species, 20µM tubulin
Distance [µm]
free tubulin and pStat gradients
normalized
tub free @stathmin 1 site
tub free @stathmin 2 sites
pStath @stathmin 1 site
pStath @stathmin 2 sites
Distance [µm]

Supplementary Fig. 6. Experimentally parametrized reaction-diffusion simulations of the stathmin-tubulin sequestration cycle

(a) Fluorescence emission spectra of 1μM COPY^{o2} (donor: Atto532, acceptor: Atto647N) in absence (solid lines) or presence of 10μM tubulin (dashed lines) upon 520nm excitation. (b) Quantification of COPY^{o2} FRET efficiency by Time-Correlated Single Photon Counting Fluorescence Lifetime Imaging Microscopy (TCSPC-FLIM). Phasor plot representation of the Fourier components of the first harmonic frequency (20MHz) of TCSPC-FLIM data is shown. Fluorescence lifetime of the COPY^{o2} construct was determined by global analysis of FLIM data. The fluorescence lifetime of COPY^{o2} without acceptor (τ_D) was 3.37±0.05ns (green). COPY^{o2} exhibited lifetimes of 2.69±0.08ns with corresponding E_{FRET}: 0.2±0.03 in the absence of tubulin (red), while tubulin binding (orange) increased the lifetimes to 3.0±0.09ns and decreased E_{FRET} to 0.11±0.03. (c-f) Binding parameters of the COPY^{o2}-tubulin interaction as measured by sensitized emission in stopped flow (Methods) from linear fits of apparent association rate constants of COPY^{o2} and COPY$^{o2\text{-}P}$ to tubulin. Two association rate constants corresponding to the two tubulin heterodimers binding sites on stathmin were determined. (g) Biexponential fits of tubulin dissociation from COPY^{o2} and (h) COPY$^{o2\text{-}P}$, obtaining two dissociation rate constants. From these rate constants (inset), corresponding affinities were calculated (right, Supplementary Table 1). (i) 1D reaction-diffusion simulations of phosphorylated stathmin (pStathmin) and tubulin (tub) gradients. The model assumes that phosphorylation-mediated release of tubulin from stathmin is mostly driven by the change in affinity of the high affinity binding site. This can be described by five interconverting species: free tub, stathmin, pStathmin, stathmin-tub and pStathmin-tub complex. Stathmin association/dissociation parameters were set to the values measured in (c-h, Methods). $k_{PP\lambda}$: dephosphorylation reaction rate constant, k_{kin}: phosphorylation reaction rate constant. Left: free tubulin (solid lines) and fraction of free pStathmin (dashed lines) versus distance to the membrane ($k_{PP\lambda}$ = 0.02s^{-1}; k_{kin} varying between 0.01 and 100s^{-1}). Inset: normalized free tubulin gradients. Right: free tubulin (solid lines) and fraction of free pStathmin (dashed lines) versus distance to the membrane (k_{kin} = 1s^{-1}; $k_{PP\lambda}$ varying between 0.001 and 10s^{-1}). Inset: normalized free tubulin gradients. (j) Left: Steady-state concentration profiles of the five reacting species (box legend) versus distance from the membrane. Right: Difference between maximum amplitude at the membrane (0μm) and at 1μm distance (Δamplitude). The amplitude difference is maximal when the kinase activity is 100 times larger than the phosphatase activity. (k) Comparison of the simplified 5-species model with a 9-species model incorporating binding/release from both tub-binding sites on stathmin. Left: spatial distribution of the species (see legend) that arise from 10μM tubulin binding to stathmin, stathmin phosphorylation in the presence of high kinase activity (k_{kin} = 10s^{-1}) at the membrane (0μm), homogenous phosphatase activity ($k_{PP\lambda}$ = 0.02s^{-1}) and stathmin association/dissociation parameters set to the values measured in (c-h, Methods). Middle: Considering an additional low affinity binding site (stat-tub01 high affinity site; stat-tub10 low affinity site; stat-tub11, both sites occupied). Right: comparing the gradient of all phosphorylated stathmin species and free tubulin for the 5- and 9-species simulation (inset: normalized tubulin gradient).

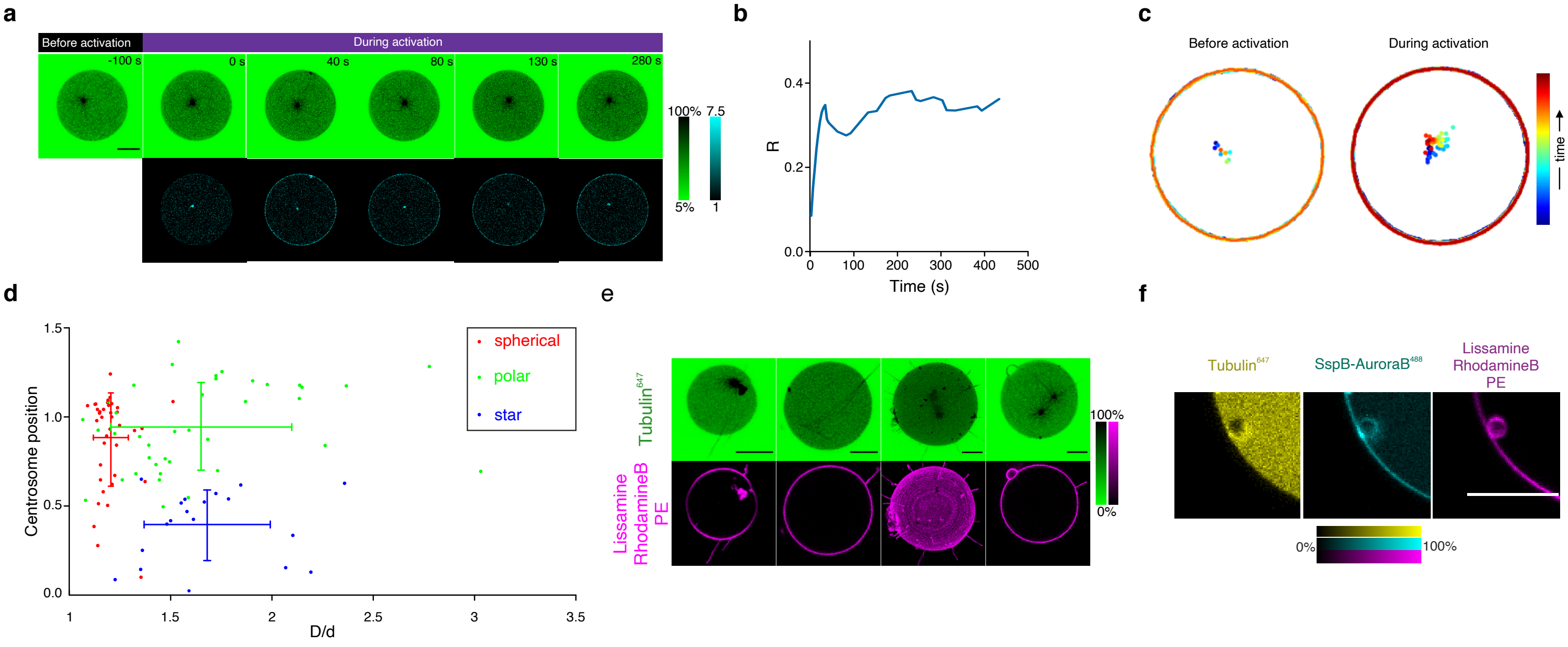

a
Before activation
During activation
-100 s
0 s
40 s
80 s
130 s
280 s
100%
5%
7.5
1
b
R
Time (s)
c
Before activation
During activation
time
d
Centrosome position
D/d
spherical
polar
star
e
Tubulin647
Lissamine RhodamineB PE
100%
0%
f
Tubulin647
SspB-AuroraB488
Lissamine RhodamineB PE
0%
100%

Supplementary Fig. 7. Control SynMMS^{-stat} with a rigid membrane, morphometric characterization of initial states, and lipid distribution around vesicles and protrusions.

(a) Selected images at indicated times of CLSM time-lapse of normalized tubulin^{647} fluorescence (upper row, inverted green color bar) and SspB-AuroraB^{488} translocation images (lower row, cyan color bar) of a SynMMS^{-stat} with a rigid membrane (iso-osmotic), before and during global 488nm irradiation. (b) Corresponding SspB-AuroraB^{488} translocation quantification (R) during activation, and (c) SynMMS^{-stat} contours and centrosome positions (dots), color-coded by time. (d) Morphometric parameters for SynMMS and encapsulated MT-aster morphologies (spherical, polar, star; one dot per GUV). 3D centrosome position (0: centered, 1: membrane proximal) versus D/d (D: diameter of minimal bounding sphere; d: diameter of maximal inscribed sphere; 1: perfect spheres; >1 for deformed shapes). Crosshairs: mean±S.D. for each morphology class. (e) CLSM normalized fluorescence images of representative SynMMS showing MT-induced protrusions with lipid tracer (top; tubulin^{647}: inverted green color bar, bottom; Lissamine-RhodamineB-PE: magenta color bar). Third SynMMS from left is a 3D projection of confocal z-stacks. (f) Representative CLSM normalized fluorescence images of a SynMMS with internalized vesicle with lipid tracer and SspB-AuroraB^{488} after light-induced membrane translocation (Left; tubulin^{647}: yellow color bar, middle; SspB-AuroraB^{488}: cyan color bar, right; Lissamine-RhodamineB-PE: magenta color bar). Tubulin^{647} fluorescence images were enhanced by histogram equalization, directional filtering and unsharp masking (methods). SspB-AuroraB^{488} translocation images were corrected for luminal fluorescence contribution (methods). Scale bars: 10μm.

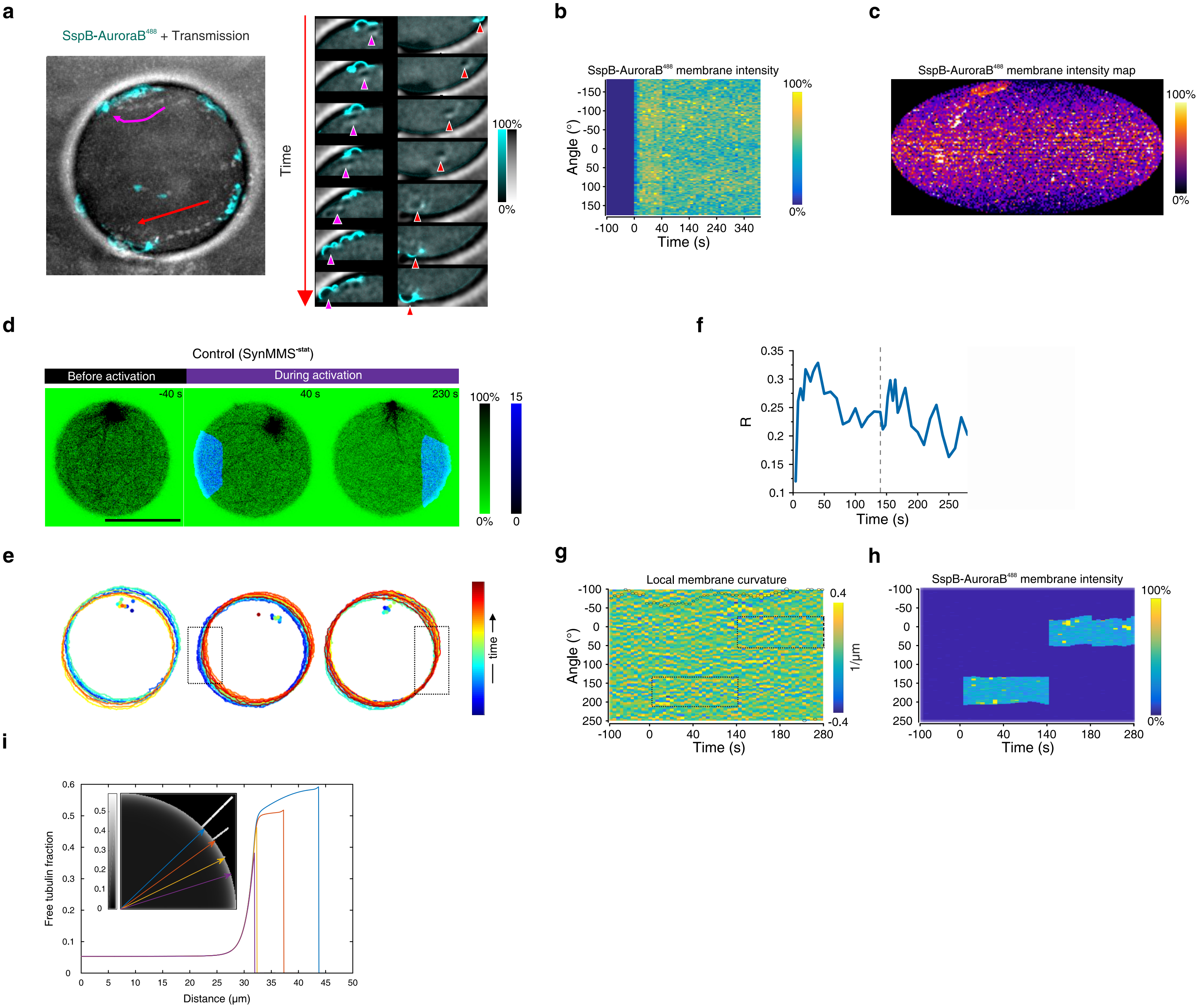

a
SspB-AuroraB488 + Transmission
Time
100%
0%
b
SspB-AuroraB488 membrane intensity
Angle (°)
Time (s)
100%
0%
c
SspB-AuroraB488 membrane intensity map
100%
0%
d
Control (SynMMS-stat)
Before activation
During activation
-40 s
40 s
230 s
100%
15
0%
0
f
R
Time (s)
e
time
g
Local membrane curvature
Angle (°)
Time (s)
0.4
-0.4
1/μm
h
SspB-AuroraB488 membrane intensity
Angle (°)
Time (s)
100%
0%
i
Free tubulin fraction
Distance (μm)

Supplementary Fig. 8. Characterization of light-induced astral-MT membrane protrusions for SynMMS and controls

(a) Left: Composite image of multiple frames (from t: [100-220s]) rendered as maximum-intensity-projections of CLSM SspB-AuroraB^{488} fluorescence and transmission images of the SynMMS in Fig. 6a. Cyan color and gray scale bars denote normalized intensity. Arrows (red and magenta) indicate movement of two different MSPs on the surface of the membrane. Right: montage of single frames from the left composite image. Colored arrowheads point at corresponding moving MSPs. (b) Angular kymograph of SspB-AuroraB^{647} membrane intensity (color bar: normalized intensity) for SynMMS^{-stat} in Fig. 6g. (c) Membrane intensity maps (color bar: normalized intensity) of translocated SspB-AuroraB^{488} after 488nm irradiation for SynMMS^{-stat} in Fig. 6g. (d) Selected images at indicated times of CLSM time-lapse of tubulin^{647} fluorescence (inverted green color bar: normalized intensity) overlaid with SspB-AuroraB^{488} translocation image (blue color bar) before and during multiple local 488nm irradiation phases of a control SynMMS^{-stat} with a sparse aster. (e) Corresponding contours and centrosome position for each phase color-coded by time (rectangles: irradiation areas), and (f) SspB-AuroraB^{488} translocation quantification (R) (dashed lines: change of irradiation phase). (g) Angular membrane curvature kymographs overlaid with centrosome position (small circle: centered, large circle: membrane proximal). Color bar: curvature as the inverse radius (1/µm) of an inscribed circle (Methods). Positive curvature: circle inside GUV; negative: circle outside. Local irradiation (dashed rectangles) starts at t=0s. (h) Corresponding angular kymograph of SspB-AuroraB^{488} membrane intensity (color bar: normalized intensity). Tubulin^{647} fluorescence images were enhanced by histogram equalization, directional filtering and unsharp masking (methods). SspB-AuroraB^{488} translocation images were corrected for luminal fluorescence contribution (methods). (i) 2D reaction-diffusion simulation of a GUV with thin protrusions illustrates the free tubulin gradient generated by stathmin phosphorylation cycle (Methods). In protrusions, the enhanced gradient amplitude further increases with their length. Colored 1D-profiles taken along lines as indicated by corresponding arrows in the inset. Scale bar: 10µm.

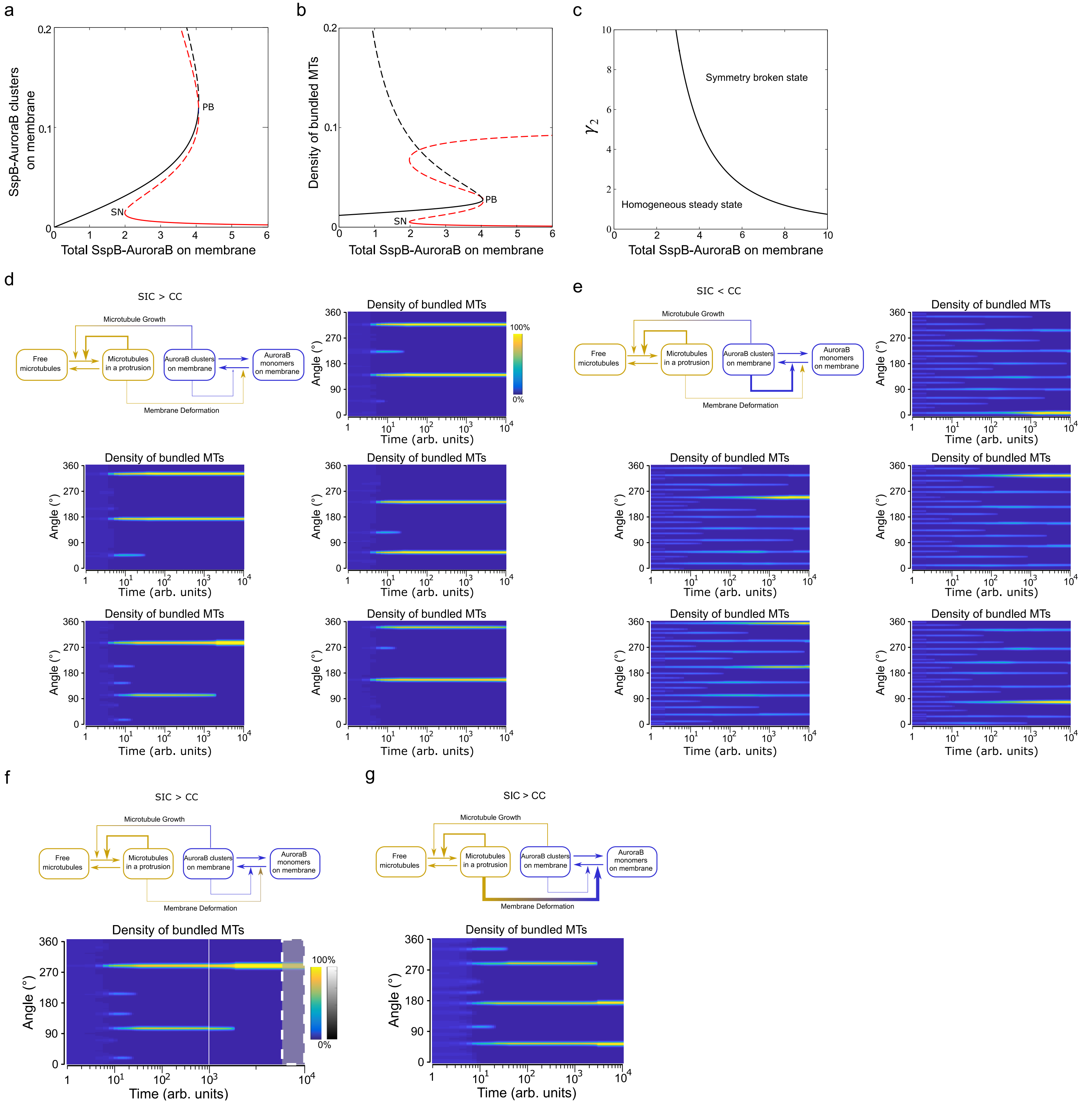
a
SspB-AuroraB clusters on membrane
Total SspB-AuroraB on membrane
PB
SN
b
Density of bundled MTs
Total SspB-AuroraB on membrane
PB
SN
c
γ_2
Symmetry broken state
Homogeneous steady state
Total SspB-AuroraB on membrane
d
SIC > CC
Microtubule Growth
Free microtubules
Microtubules in a protrusion
AuroraB clusters on membrane
AuroraB monomers on membrane
Membrane Deformation
Density of bundled MTs
Angle (°)
Time (arb. units)
100%
0%
e
SIC < CC
Microtubule Growth
Free microtubules
Microtubules in a protrusion
AuroraB clusters on membrane
AuroraB monomers on membrane
Membrane Deformation
Density of bundled MTs
Angle (°)
Time (arb. units)
f
SIC > CC
Microtubule Growth
Free microtubules
Microtubules in a protrusion
AuroraB clusters on membrane
AuroraB monomers on membrane
Membrane Deformation
Density of bundled MTs
Angle (°)
Time (arb. units)
100%
0%
g
SIC > CC
Microtubule Growth
Free microtubules
Microtubules in a protrusion
AuroraB clusters on membrane
AuroraB monomers on membrane
Membrane Deformation
Density of bundled MTs
Angle (°)
Time (arb. units)

Supplementary Fig. 9. *In silico* initial patterned states and corresponding system responses to external signals for different parameters.

Bifurcation diagram of the coupled microtubule (MT)-membrane / signaling system (Fig. 8a, equations and parameters in Methods) using (a) the density of clustered SspB-AuroraB and (b) density of bundled MTs. PB – pitchfork bifurcation; SN – saddle-node bifurcation. Solid lines: black – homogenous steady-state, red – symmetry broken state. Dashed lines: unstable steady-states. (c) Two-parameter bifurcation diagram (γ_2, total SspB-AuroraB on membrane) depicting the stability interval of the symmetry-broken solution. (d)-(g) Reaction-diffusion simulations of the coupled MT-membrane/SspB-AuroraB system (Eqs. 24, Methods). (d) Multiple realizations of stable polar initial pattern generated under random initial conditions for SIC>CC: self-amplified MT-capture (γ_1=1) dominates over SspB-AuroraB cooperative clustering (γ_2=0.5) as indicated in the top schematic. (e) Same as in (d), only for SIC<CC: SspB-AuroraB cooperative clustering (γ_2=5) dominates over self-amplified MT-capture (γ_1=1) as indicated in the top schematic. (f) Top schematic represents SIC>CC (γ_1=1, γ_2=0.5). Bottom: corresponding kymograph of density of bundled MTs showing that the polar initial state with single MT-bundle is stable upon global stimulation (dashed box). (g) Top schematic: SIC>CC (γ_1=1, γ_2=0.5), with a strongly enhanced intersystem link (k_4=10) of membrane deformations causing SspB-AuroraB clustering. Bottom: starting from random initial conditions, kymograph of density of bundled MTs shows the evolution to a polar stable state with two MT-bundles. Time scales and color bars description as in Fig. 8. Equations and remaining parameters in Methods.

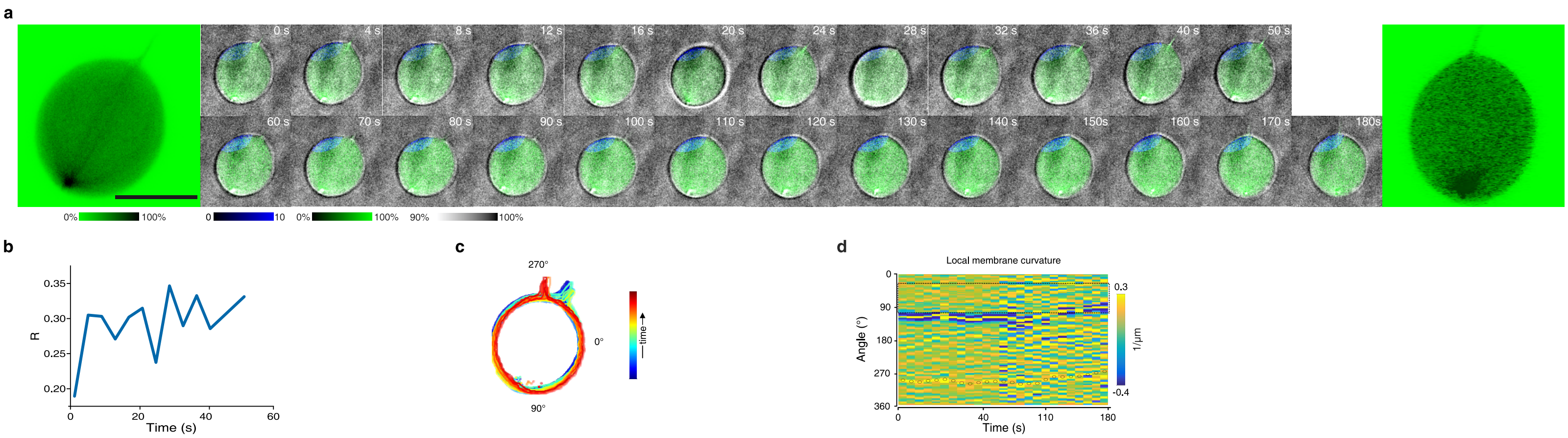
a
0 s
4 s
8 s
12 s
16 s
20 s
24 s
28 s
32 s
36 s
40 s
50 s
60 s
70 s
80 s
90 s
100 s
110 s
120 s
130 s
140 s
150s
160 s
170 s
180s
0%
100%
0
10
0%
100%
90%
100%
b
R
Time (s)
c
270°
0°
90°
time
d
Local membrane curvature
Angle (°)
Time (s)
1/μm
0.3
-0.4

Supplementary Fig. 10. Morphological changes towards local light cues depends on initial states

(a) Montage of all recorded frames of the CLSM time-lapse of a polar SynMMS (at indicated times) as an overlay of normalized tubulin^{647} fluorescence (green color bar), SspB-AuroraB^{488} translocation image (blue color bar) and normalized transmission (gray scale bar) during local 488nm irradiation, flanked by maximum-intensity-projections of normalized tubulin^{647} fluorescence CLSM z-stacks before (left) and after (right) stimulation. 650 nm excitation of Tubulin^{647} was reduced from frame 13 to minimize bleaching. (b) Corresponding SspB-AuroraB^{488} translocation quantification (R) during activation, and (c) SynMMS contours and centrosome positions (dots), color-coded by time. (d) Angular membrane curvature kymograph overlaid with centrosome position (small circle: centered, large circle: membrane proximal). Color bar: curvature as the inverse radius (1/µm) of an inscribed circle (Methods). Positive curvature: circle inside GUV; negative: circle outside. Local irradiation (dashed rectangle) starts at t=0s. Tubulin^{647} fluorescence images were enhanced by histogram equalization, directional filtering and unsharp masking (methods). SspB-AuroraB^{488} translocation images were corrected for luminal fluorescence contribution (methods). Scale bars: 10µm.

Supplementary Table 1. Kinetic parameters of stathmin phosphorylation/dephosphorylation and interaction with tubulin.

	**Stathmin**	**pStathmin**
**Phosphorylation**		
k_{cat}/K_M	$11 \pm 0.3 \cdot 10^2$ $M^{-1}s^{-1}$	
**Dephosphorylation**		
k_{cat}		0.35 ± 0.03 s^{-1}
K_M		16 ± 3 μM
k_{cat}/K_M		$22 \pm 5 \cdot 10^3$ $M^{-1}s^{-1}$
**Tubulin interaction**		
association rate constants	$k_{on1} = 16 \pm 1.6 \cdot 10^5$ $M^{-1}s^{-1}$<br>$k_{on2} = 9.0 \pm 0.1 \cdot 10^5$ $M^{-1}s^{-1}$	$k_{on1} = 12.8 \pm 0.7 \cdot 10^3$ $M^{-1}s^{-1}$<br>$k_{on2} = 17 \pm 1.3 \cdot 10^4$ $M^{-1}s^{-1}$
dissociation rate constants	$k_{off1} = 0.0480 \pm 0.0007$ s^{-1}<br>$k_{off2} = 1.86 \pm 0.01$ s^{-1}	$k_{off1} = 8 \pm 1$ s^{-1}<br>$k_{off2} = 2.3 \pm 0.1$ s^{-1}
affinities	$K_{D1} = 30 \pm 3$ nM<br>$K_{D2} = 2.00 \pm 0.02$ μM	$K_{D1} = 610 \pm 84$ μM<br>$K_{D2} = 13 \pm 1$ μM

Supplementary Table 2. Amino acid sequences of used recombinant protein constructs.

Construct details	amino acid sequence
**Gly-Stathmin-Cys**<br>pentaglycine, mouse Stathmin Δ1, additional C-terminal cysteine	GGGGGASSDIQVKELEKRASGQAFELILSPRSKESVPDFPLSPPKKKDLSLEEIQKKLEA<br>AEERRKSHEAEVLKQLAEKREHEKEVLQKAIEENNNFSKMAEEKLTHKMEANKENRE<br>AQMAAKLERLREKDKHVEEVRKNKESKDPADETEADC
**Gly-iLID-tRac1**<br>pentaglycine, SGS linker,<br>iLID, $(GS)_4$ linker,<br>C-terminus of human Rac1 (175-192)	GGGGGSGSLATTLERIEKNFVITDPRLPDNPIIFASDSFLQLTEYSREEILGRNCRFLQGPE<br>TDRATVRKIRDAIDNQTEVTVQLINYTKSGKKFWNVFHLQPMRDYKGDVQYFIGVQL<br>DGTERLHGAAEREAVCLIKKTAFQIAEAANDENYFGSGSGSCPPPVKKRKRKCLLL
**Gly-C2-iLID**<br>tetraglycine, $(GS)_4$ linker, C2 domain from bovine lactadherin (270-427), $(GS)_3$, helical $(EAAAK)_3$ linker, $(GS)_3$, iLID	GGGGGSGSGSGSCTEPLGLKDNTIPNKQITASSYYKTWGLSAFSWFPYYARLDNQGKF<br>NAWTAQTNSASEWLQIDLGSQKRVTGIITQGARDFGHIQYVAAYRVAYGDDGVTWTEY<br>KDPGASESKIFPGNMDNNSHKKNIFETPFQARFVRIQPVAWHNRITLRVELLGCGSGSG<br>SEAAAKEAAAKEAAAKGSGSGSLATTLERIEKNFVITDPRLPDNPIIFASDSFLQLTEYSR<br>EEILGRNCRFLQGPETDRATVRKIRDAIDNQTEVTVQLINYTKSGKKFWNVFHLQPMR<br>DYKGDVQYFIGVQLDGTERLHGAAEREAVCLIKKTAFQIAEAANDENYF
**λ-phosphatase**<br>PPase ORF221 of bacteriophage lambda	GMRYYEKIDGSKYRNIWVVGDLHGCYTNLMNKLDTIGFDNKKDLLISVGDLVDRGAE<br>NVECLELITFPWFRAVRGNHEQMMIDGLSERGNVNHWLLNGGGWFFNLDYDKEILAK<br>ALAHKADELPLIIELVSKDKKYVICHADYPFDEYEFGKPVDHQQVIWNRERISNSQNGI<br>VKEIKGADTFIFGHTPAVKPLKFANQMYIDTGAVFCGNLTLIQVQGEGA
**Gly-SspB-λ-phosphatase**<br>tetraglycine, $(GS)_4$,<br>SspB (H. influenza, 5-114 Y11K A15E), $(GS)_4$, PPase ORF221 of bacteriophage lambda	GGGGGSGSGSGSSSPKRPKLLREYYDWLVDNSFTPYLVVDATYLGVNVPVEYVKDGQI<br>VLNLSASATGNLQLTNDFIQFNARFKGVSRELYIPMGAALAIYARENGDGVMFEPEEIY<br>DELNIGSGSGSGSMRYYEKIDGSKYRNIWVVGDLHGCYTNLMNKLDTIGFDNKKDLLI<br>SVGDLVDRGAENVECLELITFPWFRAVRGNHEQMMIDGLSERGNVNHWLLNGGGWF<br>FNLDYDKEILAKALAHKADELPLIIELVSKDKKYVICHADYPFDEYEFGKPVDHQQVI<br>WNRERISNSQNGIVKEIKGADTFIFGHTPAVKPLKFANQMYIDTGAVFCGNLTLIQVQGE<br>GA
**Gly-SspB-AuroraB**<br>tetraglycine, $(GS)_4$, SspB (H. influenza, 5-114 Y11K A15E), $(GS)_4$, human AuroraB (45-344)<br><br>human **INCENP** (834-902)	GGGGGSGSGSGSSSPKRPKLLREYYDWLVDNSFTPYLVVDATYLGVNVPVEYVKDGQI<br>VLNLSASATGNLQLTNDFIQFNARFKGVSRELYIPMGAALAIYARENGDGVMFEPEEIY<br>DELNIGSGSGSGSMSNVQPTAAPGQKVMENSSGTPDILTRHFTIDDFEIGRPLGKGKFG<br>NVYLAREKKSHFIVALKVLFKSQIEKEGVEHQLRREIEIQAHLHHPNILRLYNYFYDRRR<br>IYLILEYAPRGELYKELQKSCTFDEQRTATIMEELADALMYCHGKKVIHRDIKPENLLLG<br>LKGELKIADFGWSVHAPSLRRKTMCGTLDYLPPEMIEGRMHNEKVDLWCIGVLCYEL<br>LVGNPPFESASHNETYRRIVKVDLKFPASVPMGAQDLISKLLRHNPSERLPLAQVSAHP<br>WVRANSRRVLPPSALQSVA<br><br>MDEAHPRKPIPTWARGTPLSQAIIHQYYHPPNLLELFGTILPLDLEDIFKKSKPRYHKRT<br>SSAVWNSPPL
