# Supplementary material for: A self-organized synthetic morphogenic liposome responds with shape changes to local light cues
Source: Nat Commun. 2021 Mar 9;12:1548. doi: 10.1038/s41467-021-21679-2 (PMC7943604; doi:10.1038/s41467-021-21679-2)
Supplement: Supplementary file 3 — Description of Additional Supplementary Files [file 41467_2021_21679_MOESM3_ESM.docx]

**Description of Additional Supplementary Files**

File Name: **Supplementary Video 1**

Description: CLSM time-lapse of tubulin^658^ (yellow) fluorescence shows MT-aster-growth induced by temperature change in a GUV with a rigid membrane (left, iso-osmotic) and a GUV with a deformable membrane (right, hyper-osmotic), corresponding to Fig. 1c,d, respectively.

File Name: **Supplementary Video 2**

Description: Monte Carlo simulations displaying global MT organization. Polar or star-like MT-bundle distributions are obtained for different total amount of MTs, left: MT_total_=20; right: MT_total_=80. Corresponding to Supplementary Fig. 1g.

File Name: **Supplementary Video 3**

Description: CLSM time-lapse of tubulin^647^ fluorescence (inverted green) overlaid with SspB-AuroraB^488^ translocation (blue) of a SynMMS with a rigid membrane (isosmotic), corresponding to Fig. 5a.

File Name: **Supplementary Video 4**

Description: CLSM time-lapse of tubulin^647^ fluorescence (inverted green) overlaid with SspB-AuroraB^488^ translocation (blue) of a SynMMS with a deformable membrane (hyper-osmotic), corresponding to Fig. 5f.

File Name: **Supplementary Video 5**

Description: CLSM time-lapse of tubulin^647^ fluorescence (inverted green) overlaid with SspB-AuroraB^488^ translocation (blue) of a SynMMS (left) and SynMMS^-stat^ (right) with deformable membranes (hyper-osmotic) and sparse MT-asters, corresponding to Fig. 6a,g, respectively.

File Name: **Supplementary Video 6**

Description: CLSM time-lapse of tubulin^647^ fluorescence (inverted green) overlaid with SspB-AuroraB^488^ translocation (blue) of a SynMMS with a deformable membrane (hyper-osmotic) and sparse aster, corresponding to Fig. 6i.

File Name: **Supplementary Video 7**

Description: Left: CLSM time-lapse of tubulin^647^ fluorescence (inverted green) overlaid with SspB-AuroraB^488^ translocation (blue) of a star-like SynMMS with isotropically distributed small protrusions. Right: Corresponding CLSM Transmission (gray scale) overlaid with its curvature contour (false color). Corresponding to Fig. 9a.

File Name: **Supplementary Video 8**

Description: Left: CLSM time lapse of tubulin^647^ fluorescence (inverted green) overlaid with SspB-AuroraB^488^ translocation (blue) of a polar SynMMS with few but strong protrusions. Right: Corresponding CLSM Transmission (gray scale) overlaid with its curvature contour (false color). Corresponding to Fig. 9j.
